# The impact of *Mycobacterium tuberculosis* complex in the environment on one health approach

**DOI:** 10.3389/fpubh.2022.994745

**Published:** 2022-09-07

**Authors:** Haobo Zhang, Mengda Liu, Weixing Fan, Shufang Sun, Xiaoxu Fan

**Affiliations:** National Animal Tuberculosis Reference Laboratory, Division of Zoonoses Surveillance, China Animal Health and Epidemiology Center, Qingdao, China

**Keywords:** *Mycobacterium tuberculosis* complex (MTBC), one health (OH), environment - agriculture, detection method, control & measurement

## Abstract

Tuberculosis caused by the *Mycobacterium tuberculosis* complex (MTBC) has become one of the leading causes of death in humans and animals. Current research suggests that the transmission of MTBC in the environment indirectly transmit to humans and animals with subsequent impact on their wellbeing. Therefore, it is of great significance to take One Health approach for understanding the role of MTBC in not only the interfaces of humans and animals, but also environment, including soil, water, pasture, air, and dust, etc., in response to the MTBC infection. In this review, we present the evidence of MTBC transmission from environment, as well as detection and control strategies in this interface, seeking to provide academic leads for the global goal of End Tuberculosis Strategy under multidisciplinary and multisectoral collaborations.

## Introduction

The *Mycobacterium tuberculosis* complex (MTBC) constitutes a significantly genetically similar group of bacteria that cause tuberculosis in a wide range of hosts. They are rod-shaped, acid-base-fast, aerobic, slow-growing intracellular pathogens that destroy phagosomal cells to maintain and evade the immune system (1). The existence of MTBC has a long history. In 2001, scientists discovered MTBC in fossil samples of the American bison palm from 17,000 years ago and found in the fossils of bighorn sheep and musk ox in the same period (2). The major MTBC pathogenic mycobacteria species include *M. tuberculosis, M. bovis, M. africanum, M. microti, M. caprae, M. pinnipedii* and *M. canettii* (3). Among them, *M. tuberculosis* is the leading infectious pathogen in the world causing tuberculosis in human, which was first discovered by Robert Koch in 1822 (4). *M. tuberculosis* is often present in the sputum of TB patients, thereby contaminating the ground and air. *M. bovis*, first identified in 1896, is the main causative bacterium of bovine tuberculosis. *M. bovis* has a wider host than *M. tuberculosis*, and in addition to cattle, it exhibits the broad spectrum of host infections, including 50 types of vertebrates and 25 birds such as badgers, possums, ferrets, red deer, and wild boar, etc. (5). Thus, infected animals can contaminate the environment through water sources, soil, etc. Except for members of *M. tuberculosis, M. bovis* found a century ago, *M. africanum* was first isolated from the lungs of African tuberculosis patients in 1968, and *M. canettii* was first reported in 1969, both of which can cause tuberculosis in humans (6). *M. microti, M. caprae*, and *M. pinnipedii* were first isolated and identified from animals. *M. microti* was first isolated and identified from voles in 1957 and *M. caprae* was first isolated from Spanish goats in 1999. They all may be infectious to humans and other wild animals (7, 8).

The pathogenesis caused by MTBC is a complex process involving complex interactions between the host immune system and bacteria. Generally, humans or animals inhale infectious droplets containing MTBC, which travel along the respiratory tract into terminal alveoli, where they are phagocytosed by alveolar macrophages and other phagocytes (9). During the initial phase of infection, MTBC is internalized by alveolar macrophages, and alveolar macrophages provide the main cellular niche for MTBC to replicate intracellularly. Thus, while protecting the host from mycobacterial invasion, macrophages also promote the establishment of early MTBC infection and maintain incubation period of infection. Bacteria-laden immune cells may be transported across the alveolar barrier to cause systemic spread (9). The most typical pathological manifestation of tuberculosis is the nodular lesions observed in lungs, which are termed tubercles. At present, tuberculosis caused by MTBC in humans and animals has been 13th cause of death and the second leading infectious killer after COVID-19 (above HIV/AIDS) worldwide, critically threatening the health of humans and animals (10).

One Health approach is to recognize that human health is closely related to the animal health, plant health and the environment they share (11). One Health approach encourages the cross of traditional boundaries of medicine and veterinary medicine. In the 19th and early 20th centuries, Louis Pasteur, and Robert Koch along with physicians like William Osler and Rudolf Virchow broke boundaries between animal and human health, which played a pivotal role on the control of infectious diseases (12). Similarly, One Health approach to tuberculosis control requires understanding, monitoring, and control of how tuberculosis spreads between humans, animals, and the environment. This highlights communication and collaboration between human health experts such as doctors, animal health experts such as veterinarians, environmental experts such as ecologists, and government and law enforcement agencies (13).

In most developed countries, tuberculosis is an almost controlled disease, but it continues to be a global challenge to the One Health due to the high burden and cost of tuberculosis in developing countries (14). In 2020, the 30 high tuberculosis burden countries accounted for 86% of new tuberculosis cases. Eight countries account for two thirds of the total, with India leading the count, followed by China, Indonesia, the Philippines, Pakistan, Nigeria, Bangladesh, and South Africa. In 2020, WHO reported that there were 10 million people infected with tuberculosis and 1.5 million died (including 214 000 people with HIV) (10). Meanwhile, due to close interaction between human and animals, other MTBC species, especially *M. bovis*, pose a zoonotic challenge to the One Health. Although some countries declare the eradication of bovine tuberculosis, *M. bovis* is still widespread in most of the developing countries (15). It has been estimated that the global prevalence rate of *M. bovis* infection in human cases is 12.1% during 2009–2019 (16).

**Figure 1:**
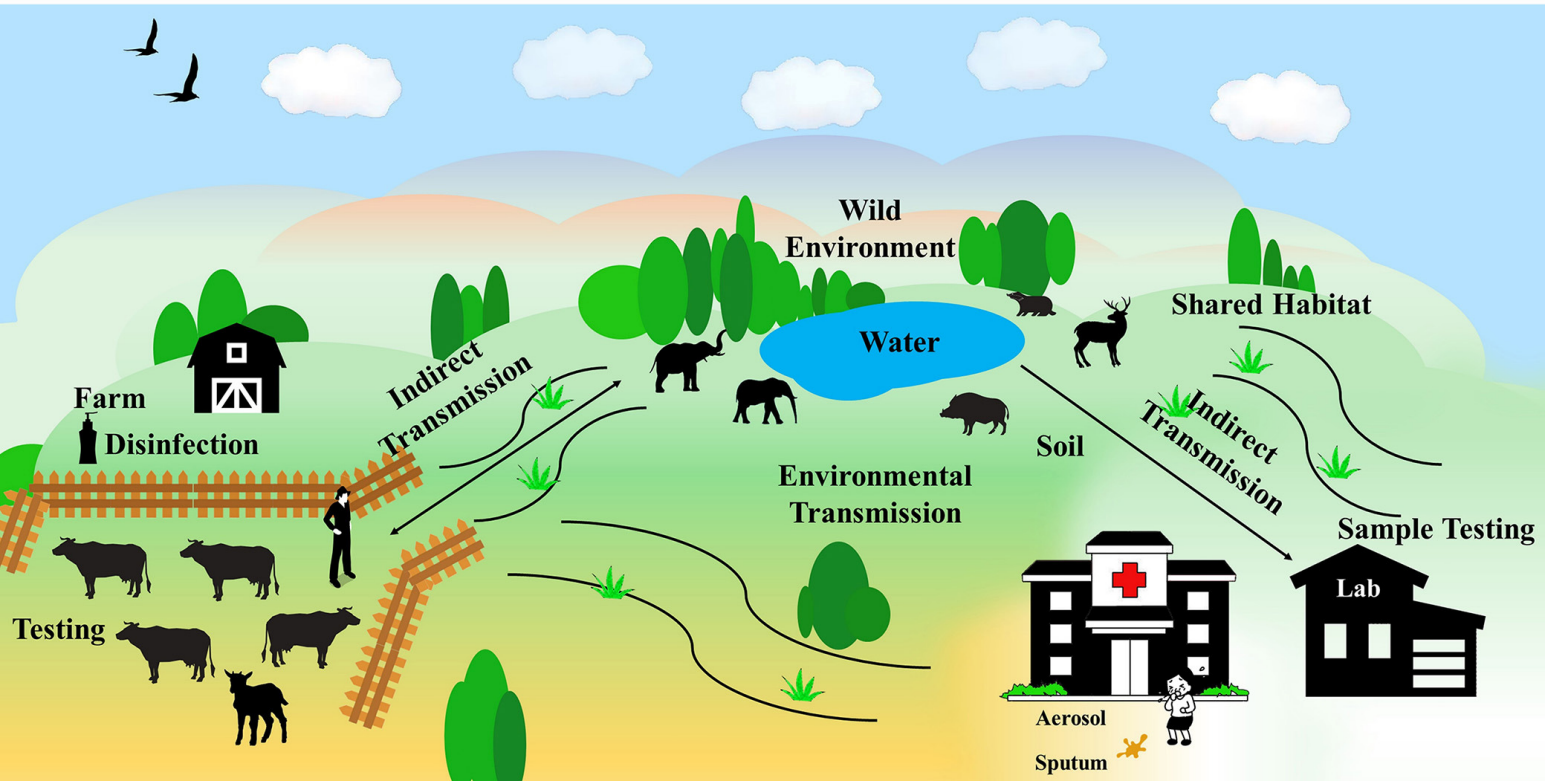
The spread of MTBC in the environment. MTBC spreads among farms, wild environment, and human habitats, causing tuberculosis infection in humans and animals.

Human infection with tuberculosis is mainly by inhalation of contaminated droplets from patients and environment, though *M. bovis* infection can be transmitted *via* uncooked meat, unpasteurized milk or occasionally from direct contact with infected livestock or offal (17). Environmental contamination of MTBC is somehow implicated to the indirect tuberculosis transmission to humans and animals. It is unraveled that MTBC has been widespread in the environment, including soil, water, pasture, air, and dust (18). Markedly, according to experimental studies, MTBC bacteria exhibit strong tolerance for harsh environment for even months (19). Therefore, the contamination of the MTBC may pose a huge threat to not only the environment, but also linked humans and animals. In this scenario, this minireview summaries the impacts of the MTBC environmental contamination on the tuberculosis infection of humans and animals and provides feasible ways for the detection and control of the etiologic agents (Figure 1).

## The *Mycobacterium tuberculosis* complex in the environment infects humans and animals worldwide

A multitude of evidence demonstrate that MTBC has been detected from many environmental samples around the world (19–21), which suggests that MTBC in the environment, such as water sources, soil, etc., represents a potential source of infection detrimental to people and animals. MTBC are released from infected individuals to the environment *via* sputum, feces, urine, aerosols, etc. (22, 23). Due to the typical structure of mycobacteria rich in lipids in cell wall, the strong tolerance of MTBC benefits the survival for 10 months in dry sputum and 5 months in water (24). The long-term viability of MTBC in the environment increases the risk of exposure to a variety of species sharing the same habitat.

In Asia, captive elephants and wild elephants come into contact when breeding, or share feeding and watering areas. Tuberculosis cases have been confirmed among captive elephants in Nepal (25), and it is found that 24% of 250 captive elephants were serologically positive (26). Similarly in Thailand, *M. tuberculosis* isolated from captive elephants was sequenced and strains appeared to have genetically originated from humans (27), indicating their living environment is inevitably contaminated. When captive elephants and wild elephants are exposed to the same contaminated environment, the risk of tuberculosis infection dramatically increases. Since humans and elephants share the same habitat, the contamination of MTBC in the environment may be one of the reasons for the high epidemic of human tuberculosis among elephant handlers.

Similarly in Europe and USA, Barasona et al. focus on the habitat of cattle, wild boar, and red deer in Spain and Portugal, where tuberculosis is highly prevalent. It is noted that 55.8% of the water points for animal, such as small waterholes, were tested positive for MTBC in mud samples on the shore, while 8.9% of them were positive in the case of water samples (19). This suggested water points contributes to potential critical risks for MTBC transmission. In Italy, Tagliapietra et al. investigated the presence of MTBC in wild rodents, water or soil samples collected at wild boar habitat. Of note, the isolates with similar genetic profile were found from both wild rodents and water samples, which were strikingly linked to those previously isolated from wild boars (28). Their study suggested that contaminated environment, such as at animal aggregation sites, played pivotal roles in the infection of MTBC in wild rodents and wild boars. In the United Kingdom and Ireland, researchers used GPS collars on cattle and badgers to track their activities and found that the direct contact between cattle and wild animals is actually fairly limited, indicating that direct transmission of tuberculosis may not be the main mechanism of interspecies transmission, implying the elevating possibility of indirect transmission through a contaminated shared environment (29, 30). In Michigan US, there is evidence that MTBC in the environment of feed contaminated by wild deer suffering from tuberculosis can also cause tuberculosis in cattle, and this has been further confirmed in the laboratory (31).

In Africa, such as Tanzania and Niger, MTBC was also detected in cattle and goat feces, soil, water, dam sediment and household dust (32), and some detected lineages were identical to the dominant *M. tuberculosis* lineages in patients, which highlights the risk of transmission from the soil to human (33).

In addition to wild environment, built environment including hospitals, nursing homes, clinics and houses are also vulnerable places to contract MTBC from contaminated dust, sputum, or common household objects. Studies document that inoculating guinea pigs with dust collected from tuberculosis wards can cause corresponding infection in guinea pigs, which provide empiric evidence that dust in tuberculosis wards has a high risk of tuberculosis for human (34). Other studies indicate that dried sputum from a tuberculosis patient placed on a handkerchief, or a blanket for 70 days still lead to tuberculosis infection in guinea pigs (35), which means that MTBC settled on some common household objects could be re-aerosolized and become a potential source of infection. Smith et al. suggested that domestic cats contracted MTBC when hunting voles, which may be infected MTBC in the environment, increasing the risk of transmission to humans (36). All the collected data confirmed that MTBC are circulating among humans, animals, and the environment they share, which make the One Health approach more difficult to be accomplished worldwide.

## Detection of *Mycobacterium tuberculosis* complex from environmental samples

It is indicated that susceptible animals can become infected in merely 3 weeks of exposure to MTBC-infected pastures (37). The detection of the MTBC in the environment helps to understand, control, and eradicate tuberculosis, and is of great significance for the endorsement of One Health approach. However, due to influencing factors such as different pretreatment methods for diverse kinds of environmental samples, along with typical reagents and sample collection methods employed, there is a lack of standardized and high-precision methods to detect MTBC in the environment. The existing methods for detecting the MTBC in the environment mainly fall into four categories: microscopic examination, culture-based methods, molecular biology methods, and whole-genome sequencing (WGS) methods.

Most of the environmental samples in the built environment are sputum, feces, and fomites from patients, while in the field environment the typical samples are mainly implicated to water, soil, and aerosols. For samples such as sputum and soil, after treatment with normal saline or PBS, microscopic examination can be performed for direct smears. In terms of water and air samples, filter collection is required before microscopic examination. Microscopic examination of MTBC is usually demonstrated with the classic Ziehl-Neelsen stain (38), but fluorescent acid-fast staining may also be used (39).

The culture method is considered the gold standard for the identification of MTBC, and environmental samples need to be pre-treated before culture to remove impurities and other bacteria. First wash with PBS and detergents such as 0.375–0.75% hexadecyl pyridinium-chloride HPC, 2–4% sodium hydroxide or 5% oxalic acid. The mixture is shaken at room temperature for 10–15 min, then neutralized. The suspension is centrifuged and discard the supernatant, and then the pellet is used for culture. For primary isolation, the pellet is usually inoculated onto a set of solid egg-based media, such as Lowenstein-Jensen; these media should contain pyruvate or pyruvate and glycerol. Agar-based media such as Middlebrook 7H10 or 7H11 or blood-based agar media can also be selected (40). Cultures were incubated at 37°C for at least 8 weeks. Some hospitals and veterinary laboratories routinely adopt liquid culture systems in which growth is measured by radiometric or fluorometric methods, such as mycobacterial growth indicator tube (MGIT) culture. The culture method proved to be the reliable, but the culture method is relatively time-consuming, and the culture of MTBC needs to be carried out in a biosafety level 3 laboratory.

Polymerase chain reaction (PCR) and real-time PCR (RT-PCR) for rapid identification of MTBC targeting 16S rRNA, insert sequences IS6110 and IS1081, and genes encoding MTBC-specific proteins, such as MPB70 have been applied (41, 42). Several commercially available kits and various “in-house” methods have been involved in the detection of MTBC in the environment, such as semiautomated PCR test GeneXpert which are effective in reducing cross-contamination of samples. These methods have been shown to be rapid and highly sensitive, however, due to the complex contents in environmental samples as well as a relatively small amount of MTBC in the environment itself, PCR methods suffer from variability and low reproducibility (43). In addition, PCR-based assays are unable to distinguish live from dead bacteria. In response to this limitation, many studies have tried RNA molecules (especially 16S RNA) or selective intracellular DNA detection to solve this problem (44, 45).

Specific identification and genotyping of MTBC are considered important means for understanding transmission among humans, animals, and the environment. Various genotyping techniques have been developed to distinguish MTBC isolates from shared environment. These methods can further identify different MTBC strains and will enable characterize the origin, spread and mode of transmission of MTBC (46). The most widely used methods are spoligotyping and 24-loci MIRU-VNTR typing. Spoligotyping can distinguish strains belonging to the interior of MTBC and MIRU-VNTR typing will enable significantly enrich our knowledge of MTBC phylogeny and global spread. However, in some cases, their discriminating power for prospective epidemiological investigations may be restricted.

The rapid development of whole-genome sequencing (WGS) including next-generation sequencing and long-read technology sequencing has made it possible to identify genetic information of MTBC in environmental samples. WGS can sequence complete genomes of multiple strains simultaneously, saving time, streamlining workflow, and provide more information than traditional methods (47). WGS resequencing allows to identify polymorphisms in MTBC based on known reference genomes, facilitating molecular epidemiological investigations of MTBC in the environment. Several studies have used WGS in epidemiological surveys, shown that WGS provides better discriminative performance and improved accuracy compared to traditional methods (48, 49). Importantly, by directly analyzing the MTBC genome, it potentially offers a cost-effective way for public health teams. In the future, WGS is promisingly as one of the main methods to detect MTBC in the environment.

## Environmental control measures for *Mycobacterium tuberculosis* complex

Minimizing the MTBC in the environment using One Health approach helps protect human and animal health. Establishing an effective disinfection system is the key to controlling MTBC in the environment. As for indoor environments such as hospitals and prisons, aerosols are still the major way to spread tuberculosis. The primary mean of air disinfection is natural ventilation which has the advantage of wide availability, low cost, and high efficacy. In 10 studies evaluating environmental interventions, including 31,776 human participants, mechanical ventilation reduced infections by 2.9 to 14% (50). However, ventilation depends on the optimal climates (51), upper-room ultraviolet germicidal irradiation (UVGI) can be supplemented as additional intervention in areas where adequate ventilation is difficult to achieve (52). The evaluation of the working parameters of the UVGI system clearly shows that the UVGI system can kill or inactivate airborne MTBC and significantly enhance the protection of healthcare workers.

In pastures, especially cattle farms, there is high possibility of the contamination with feces, droplets, and nasal mucus. This untreated manure potentially increases the risk of indirect transmission of tuberculosis (53). Therefore, comprehensive implementation of disinfection is an effective way to control MTBC in the environment (43). For the entire farm, two or three kinds of disinfectants, such as ethyl alcohol and sodium hypochlorite, are utilized alternately to thoroughly disinfect the grounds, shelters, appliances, imports and exports, vehicles, excrement, etc., which can cut off the infection routes and prevent the spread of MTBC in animal facilities.

Routine disinfection is also essential to control the spread of MTBC from environment to humans and animals. A permanent disinfection channel should be set up at high-risk regions, particularly, such as the entrance of the farm, so that personnel, vehicles, and materials can be appropriately disinfected especially when entering and leaving the farm. Effective disinfectant, such as 20% lime milk, can be applied in the disinfection channel. To ensure the actual effect of the disinfectant, replacement is required every 15 days. The surrounding environment of the cowshed (including the sports field) shall be disinfected with 2% sodium hydroxide or sprinkled with quicklime once a week. Sewage tanks, cesspits, and sewer outlets shall be disinfected with bleaching powder once a month. All utensils in the barn should be disinfected regularly. The delivery room should be disinfected once a week besides immediate disinfection before and after delivery. Milking staff, milking machines and other tools must be cleaned and disinfected every time. Additionally, it should be noted that contamination of milk by disinfectants may occur during environmental disinfection in the milking parlor.

The environment contamination by wild animals may also play an important role in tuberculosis epidemiology. It is believed transmission and infection occur through the shedding of infectious bacilli of animals, and this phenomenon is well-described in badgers, wild boars, and deer (23, 54, 55). However, eradication of MTBC in wild environment seems to be extremely impossible. In the United Kingdom, immunization of badgers with the BCG vaccine provides immune protection to badgers and is an option for reducing MTBC in the environment, playing a role in the transmission of MTBC at the wildlife and livestock interface. Another effective measure is to prevent wild animals from contaminating the living environment shared with humans and domestic animals. For example, feasible measures include the use of anti-badger fencing, raised outdoor water and feed troughs which preclude access for badgers (56).

## One health prospects

Currently, human and animal tuberculosis control programs have been implemented under normal conditions. Under the long-term efforts of WHO, OIE, FAO and the European Union, the joint aim is to reduce the percentage of tuberculosis deaths by 95% and the incidence of tuberculosis by 90% by 2035 compared with 2015 (57). However, the continued presence of MTBC in the environment is a challenge to One Health, making it a potential barrier to the 2035 target. Meanwhile, the treatment of tuberculosis acquired from the environment and the monitoring of tuberculosis in the environment also impose a huge burden on the economy. Research on tuberculosis transmission has mainly focused on the transmission between hosts, while epidemiological investigations of MTBC in the environment are less addressed. However, many studies and animal experiments have shown that MTBC exists in both natural and indoor environments, and MTBC in some of these environments, may still be contagious (58). This leads to a potentially underestimated MTBC burden in the environment, which is one of the reasons for neglect in environmental MTBC.

Therefore, One Health approach enhances the awareness of the prevention and control of MTBC in the environment, demonstrating an essential way to the goal of End Tuberculosis Strategy (59). On the one hand, medical staff and animal husbandry practitioners abide by relevant regulations and laws on epidemic prevention in the process of medical treatment, breeding, and sales, and do a good job in environmental disinfection to avoid the spread of MTBC (50). On the other hand, the government and tuberculosis prevention and control institutions should enhance the subjective perception that MTBC in the environment harms the human and animal health and pay adequate attention to the MTBC in the environment causing tuberculosis (60). At the national level, the government needs to establish a feasible MTBC monitoring and prevention system in the environment. The health department should closely monitor the disinfection records of medical and health institutions, and the animal epidemic prevention department should closely monitor the disinfection situation of farms and other environments, conduct joint monitoring of key areas, and attach attention to the prevalence and spread of MTBC among animals and humans in the environment (61).

At the national level, the government needs to initiate MTBC monitoring and prevention system in the environment. The health department should closely monitor the disinfection records of medical and health institutions. In addition to the harm of MTBC infection itself, in a high-risk environment contaminated by MTBC, HIV co-infection is the most important risk factor for the development of latent tuberculosis into active tuberculosis (62). In the meantime, MTBC infection from the environment can also negatively affect the immune response to HIV, accelerating the progression of HIV infection to AIDS (63). Animal epidemic prevention departments should closely monitor the disinfection of farms and other environments. MTBC in the environment will not only threaten animal health, but also be directly or indirectly exposed to antibiotics during treatment. It should be noted that drug-resistant MTBC pose an uncontrollable threat to the treatment and decontamination of tuberculosis (64). Joint monitoring of health and animal epidemic prevention departments should be carried out in key areas contaminated by MTBC, and the prevalence and spread of MTBC among animals and humans in the environment should be closely monitored.

For tuberculosis institutes and laboratories, it is necessary to strengthen scientific research on MTBC in the environment, develop molecular biology detection technologies, and improve the accuracy and timeliness of MTBC detection in the environment (65). Establish a comprehensive MTBC surveillance system in the environment, promote the development of molecular epidemiology, collect comprehensive and credible epidemiological data, to determine the transmission route of MTBC, and control the mutual infection of MTBC in humans, livestock, and wild animals.

## Conclusions

There is already substantial evidence of MTBC contamination in indoors, farms, and natural environments, with the potential to infect humans and animals. However, studies on tuberculosis transmission have mainly focused on direct transmission, and the potential impact of contamination of MTBC in the environment is frequently neglected. Additionally, the inappropariate detection and disinfection methods even prolong the persistence of MTBC in the environment. This review underscored the integration of environmental MTBC into tuberculosis control program, which relies on multidisciplinary and multisectoral collaborations. Also, it is of necessity for the endorsement and input of governments on surveillance, academic research, tuberculosis control products R&D, public awareness comprehensively to initiate the national and international programs at human-animal-environment interface by utilizing One Health approach to reduce the tuberculosis burden.

## Author contributions

HZ wrote the article. ML was responsible for finding references. WF and SS provided their detailed and constructive comments. XF revised the article. All authors contributed to the article and approved the submitted version.

## Funding

This work was supported by China Agriculture Research System (CARS-36).

## Conflict of interest

The authors declare that the research was conducted in the absence of any commercial or financial relationships that could be construed as a potential conflict of interest.

## Publisher's note

All claims expressed in this article are solely those of the authors and do not necessarily represent those of their affiliated organizations, or those of the publisher, the editors and the reviewers. Any product that may be evaluated in this article, or claim that may be made by its manufacturer, is not guaranteed or endorsed by the publisher.
